# Disparities in cardiovascular disease prevalence among middle-aged and older adults: Roles of socioeconomic position, social connection, and behavioral and physiological risk factors

**DOI:** 10.3389/fcvm.2022.972683

**Published:** 2022-10-14

**Authors:** Ji Zhang, Yian Fang, Yao Yao, Yang Zhao, Dahai Yue, Meekang Sung, Yinzi Jin, Zhi-Jie Zheng

**Affiliations:** ^1^School of Population Medicine and Public Health, Chinese Academy of Medical Sciences and Peking Union Medical College, Beijing, China; ^2^Department of Global Health, School of Public Health, Peking University, Beijing, China; ^3^Department of Health Policy and Management, School of Public Health, Peking University, Beijing, China; ^4^China Center for Health Development Studies, Peking University, Beijing, China; ^5^The George Institute for Global Health, University of New South Wales, Sydney, NSW, Australia; ^6^The George Institute for Global Health China, Beijing, China; ^7^Department of Health Policy and Management, School of Public Health, University of Maryland, College Park, MD, United States; ^8^Seoul National University, Seoul, South Korea; ^9^Institute for Global Health and Development, Peking University, Beijing, China

**Keywords:** prevalence, risk factors, country-level disparity, dominance analysis, cardiovascular disease(s)

## Abstract

**Introduction:**

Cardiovascular disease (CVD) remains the leading cause of premature death globally and a major contributor to decreasing quality of life. In the present study, we investigated the contribution of social, behavioral, and physiological determinants of CVD and their different patterns among middle-aged and older adults.

**Methods:**

We used harmonized data from 6 nationally representative individual-level longitudinal studies across 25 countries. We restricted the age to ≥50 years and defined cases as a self-reported history of CVD. The exposure variables were the demographic status (age and sex), socioeconomic position (education level, employment, and household income level), social connections (marital status and family size), behavioral factors (smoking, alcohol drinking, and frequency of moderate to vigorous physical activity), and physiological risk factors (obesity, presence of hypertension, and presence of diabetes). Mixed logistic regression models were fitted to investigate the associations, and dominance analysis was conducted to examine the relative contributions.

**Results:**

In total, 413,203 observations were included in the final analysis, with the CVD prevalence ranging from 10.4% in Mexico to 28.8% in the United States. Physiological risk factors were the main driver of CVD prevalence with the highest dominance proportion, which was higher in developing countries (China, 57.5%; Mexico, 72.8%) than in developed regions (United States, England, 10 European countries, and South Korea). Socioeconomic position and behavioral factors also highly contributed but were less significant in developing countries than in developed regions. The relative contribution of socioeconomic position ranged from 9.4% in Mexico to 23.4% in the United States, and that of behavioral factors ranged from 5.7% in Mexico to 26.1% in England.

**Conclusion:**

The present study demonstrated the different patterns of determinant contributions to CVD prevalence across developing and developed countries. With the challenges produced by different risk factors, the implementation of tailored prevention and control strategies will likely narrow disparities in the CVD prevalence by promoting health management and enhancing the capacity of health systems across different countries.

## Introduction

Cardiovascular disease (CVD) remains the leading cause of premature mortality and is a major contributor to reduced quality of life and rising healthcare costs globally (1). From 1990 to 2019, the total number of cases of CVD nearly doubled from 271 million to 523 million, and this rise was accompanied by a steady increase in deaths (2). Middle-aged and older adults are among the most vulnerable populations and have the largest CVD burden (3), which hinders the achievement of healthy aging and causes significant direct and indirect economic hardships (4).

Recent analyses have demonstrated inequalities in the CVD prevalence across countries and regions, and prior studies investigated the associations of social, behavioral, and physiological risk factors individually (5–7), but rarely in the same context. Identifying the relative contributions of different risk factors can elucidate the mechanisms underlying social and environmental gradients and inform policy-making in CVD prevention and control. Most of the currently available data are derived from developed countries, whereas findings from developing countries are conflicting (8–12). Developing countries bear more than 80% of the global burden of CVD, and the gap between developing and developed countries is widening (13). Comparison among countries with varying developmental levels is a necessary step toward understanding how different risk factors shape the patterns of the CVD prevalence, and it can provide implications for multilateral global health actors to identify opportunities to reduce health inequalities by multi-sectoral cooperative activities.

To fill these research gaps, we investigated the contributions of socioeconomic position, social connection, and behavioral and physiological factors to the CVD prevalence among middle-aged and older adults in a series of unified and standardized population-based longitudinal studies conducted across 25 countries on 3 continents—North America, Europe, and Asia. Given that social and environmental risk factors for CVD are a multidimensional construct that largely depends on the context, we demonstrated the different patterns of determinant contributions across settings.

## Methods

### Data source

This study included six individual-level cohorts on aging that used modules similar to that of the Health and Retirement Study (HRS) in the United States (14), with harmonized data provided to facilitate the cross-national study of aging (https://g2aging.org/) (15). The HRS family surveys draw a nationally representative sample of the country's older population and are designed as longitudinal surveys with the goal of re-interviewing the same individuals on an approximately biennial basis. To maintain a nationally representative sample over time, periodic refresher samples bring in newly age-eligible respondents, although the frequency of refresher samples varies across surveys (15).

In this study, we obtained data from six family studies: the HRS; the Survey of Health, Aging and Retirement in Europe (SHARE) for 10 European developed countries (Austria, Belgium, Czech Republic, Denmark, France, Germany, Italy, Spain, Sweden, and Switzerland); the English Longitudinal Study of Aging (ELSA) in England; the Korean Longitudinal Study of Aging (KLoSA) in South Korea; the China Health and Retirement Longitudinal Study (CHARLS) in China; and the Mexican Health and Aging Study (MHAS) in Mexico. We categorized the included countries' level of development according to the income level from the World Bank database (https://data.worldbank.org/). Specifically, we included 13 developed countries (the United States, England, Austria, Belgium, Czech Republic, Denmark, France, Germany, Italy, Spain, Sweden, Switzerland, and South Korea) and 2 developing countries (China and Mexico).

Different waves were selected from each survey for a similar time range: HRS, 2010–2018; ELSA, 2010–2018; SHARE, 2011–2017; KLoSA, 2011–2019; CHARLS, 2011–2018; and MHAS, 2012–2018. We excluded participants aged <50 years and those with missing outcomes and exposures, and we finally enrolled 93,945 participants from HRS, 35,706 participants from ELSA, 178,133 participants from SHARE, 37,503 participants from KLoSA, 35,791 participants from CHARLS, and 32,125 participants from MHAS (Supplementary Table 1).

### Measures

#### CVD

CVD was defined as a combination of a doctor-diagnosed heart problem and doctor-diagnosed stroke, but slight differences exist in phrasing across studies. The concordance across studies is shown in Supplementary Table 2. Respondents were asked whether they had had the condition since the last interview starting in the second wave. According to their responses, imputations were made by both the team of the gateway to global aging data and our team to obtain a correct report in each wave.

#### Explanatory variables

Five groups of risk factors were identified in this study: demographic status (age and sex), socioeconomic position (education level, employment, and household income level), social connections (marital status and family size), behavioral factors (smoking, alcohol drinking, and frequency of moderate to vigorous physical activity), and physiological risk factors (obesity, presence of hypertension, and presence of diabetes). All the variables are categorical, and detailed scales and cutoffs are listed in Supplementary Table 3.

### Statistical analyses

To investigate the association between risk factors and CVD prevalence, we fitted mixed logistic models with repeated observations of individuals separately in each study. Unstructured mixed logistic modeling was used due to the nominal nature of the dependent variables. To account for clustering, a multilevel approach was adopted, and repeated individuals were fitted as the random effects. Regression coefficients and prospective odds ratios (with 95% CIs) in the logistic regression were calculated. To quantify the extent to which a risk factor was associated with the CVD prevalence, we conducted a dominance analysis for decomposition by examining the relative importance of explanatory variables that contributed to the R-squared of the regressions. All analyses were conducted using Stata 14.1 (StataCorp LP, College Station, TX, USA).

## Results

### Study sample characteristics

Table 1 shows the characteristics of the participants in each cohort. The prevalence of CVD in the HRS, ELSA, SHARE, KLoSA, CHARLS, and MHAS was 28.77, 23.53, 20.21, 13.14, 20.57, and 10.39%, respectively.

**Table 1:** Variables included in the present analysis with summary statistics by cohort (country/region).

	**HRS**		**ELSA**		**SHARE**		**KloSA**		**CHARLS**		**MHAS**	
	**N**	**%**	**N**	**%**	**N**	**%**	**N**	**%**	**N**	**%**	**N**	**%**
Total N	93,945		33,963		129,700		37,503		35,791		181,26	
Diagnosed with Cardiovascular Diseases	27,025	28.77	7,990	23.53	26,214	20.21	4,929	13.14	7,363	20.57	1,883	10.39
**Demographic**												
Female gender	54,509	58.02	18,533	54.57	70,104	54.05	21,439	57.17	18,774	52.45	10,804	59.6
Average age (means, std)	67.28, 0.04		67.50, 0.05		67.20, 0.03		67.32, 10.39		62.19, 8.33		67.06, 9.36	
Age ≥70 years at interview	38,022	40.47	13,774	40.56	50,692	39.08	15,614	41.63	6,485	18.12	7,004	38.64
**Social connection**												
Marital status												
Married/partnered	57,108	60.79	24,462	72.03	93,866	72.37	28,576	76.2	30,814	86.09	11,825	65.24
Living alone	31,574	33.61	7,786	22.92	29,409	22.67	8,635	23.02	4,695	13.12	5,490	30.29
Never married	5,263	5.6	1,715	5.05	6,425	4.95	292	0.78	282	0.79	811	4.47
Family Size (number of people living in household)												
1	22,738	24.2	7,877	23.19	31,407	24.22	2,818	7.51	1,960	5.48	5,812	32.06
2	44,283	47.14	20,657	60.82	74,576	57.5	14,469	38.58	14,219	39.73	6,542	36.09
≥3	26,924	28.66	5,429	15.99	23,717	18.29	20,216	53.91	19,612	54.8	5,772	31.84
**Socioeconomic position**												
Education level												
Less than lower secondary	17,133	18.24	9,102	26.8	51,673	39.84	21,927	58.47	32,240	90.08	15,780	87.06
Upper secondary & vocational	31,154	33.16	17,755	52.28	48,330	37.26	11,669	31.11	3,082	8.61	610	3.37
Tertiary	45,658	48.6	7,106	20.92	29,697	22.9	3,907	10.42	469	1.31	1,736	9.58
Currently working for pay	36,567	38.92	10,551	31.07	40,946	31.57	15,259	40.69	25,221	70.47	6,648	36.68
Household income level^*^
Lower tertile	31,315	33.33	11,321	33.33	43,725	33.71	13,203	35.21	11,970	33.44	6,034	33.29
Intermediate tertile	31,316	33.33	11,389	33.53	44,071	33.98	11,725	31.26	11,888	33.22	6,063	33.45
Highest tertile	31,314	33.33	11,253	33.13	41,904	32.31	12,575	33.53	11,933	33.34	6,029	33.26
**Behavioral Factors**												
Smoking												
Never	41,817	44.51	12,854	37.85	67,452	52.01	25,975	69.26	20,773	58.04	12,215	67.39
Currently smoking	13,092	13.94	3,538	10.42	23,773	18.33	5,040	13.44	10,020	28	2,344	12.93
Former	39,036	41.55	17,571	51.74	38,475	29.66	6,488	17.3	4,998	13.96	3,567	19.68
Alcohol Drinking	51,945	55.29	29,417	86.61	97,557	75.22	12,776	34.07	14,216	39.72	4,320	23.83
Frequency of physical activity												
Never	20,312	21.62	5,382	15.85	13,639	10.52	24,789	66.1	13,589	37.97	11,772	64.95
1-3 per month	10,359	11.03	23,580	69.43	6,317	4.87	NA	NA	678	1.89	6,354	35.05
≥1 per week	63,274	67.35	5,001	14.72	10,9744	84.61	12,714	33.9	21,524	60.14	NA	NA
**Physiological risk factors**												
Obesity
Normal	28,104	29.92	13,495	39.73	51,954	40.06	28,966	77.24	26,134	73.02	6,890	38.01
Overweight	33,790	35.97	11,743	34.58	52,005	40.1	8,065	21.5	8,122	22.69	6,908	38.11
Obesity	32,051	34.12	8,725	25.69	25,741	19.85	472	1.26	1,535	4.29	4,328	23.88
Average Body Mass Index(means, std)	29.92, 0.03		28.23, 0.03		26.70, 0.01		23.25, 0.01		24.34, 0.15		27.54, 0.04	
Presence of hypertension	57,251	60.94	14,701	43.29	61,677	47.55	15,250	40.66	12,392	34.62	8,746	48.25
Presence of diabetes	23,783	25.32	3,864	11.38	19,288	14.87	6,646	17.72	3,585	10.02	4,689	25.87
Wave												
Wave 1	20,566	21.89	7,427	21.87	28,622	22.07	7,648	20.39	8,188	22.88	11,978	66.08
Wave 2	19,503	20.76	7,855	23.13	34,115	26.3	7,484	19.96	5,440	15.2	6,148	33.92
Wave 3	17,895	19.05	6,748	19.87	35,781	27.59	7,948	21.19	9,822	27.44	NA	NA
Wave 4	19,651	20.92	6,333	18.65	31,182	24.04	7,485	19.96	12,341	34.48	NA	NA
Wave 5	16,330	17.38	5,600	16.49	NA	NA	6,938	18.5	NA	NA	NA	NA

HRS, Health and Retirement Study; ELSA, English Longitudinal Study of Aging; SHARE, Survey of Health, Aging and Retirement in Europe; KLoSA, Korean Longitudinal Study of Aging; CHARLS, China Health and Retirement Longitudinal Study; MHAS, Mexican Health and Aging Study; std, standard deviation; NA, not available.

* Household income level was divided into highest, intermediate, and lowest tertiles within countries.

The proportions of female and advanced-age patients were lowest in CHARLS (52.45 and 18.12%, respectively). The family size of the older population was larger than three in KLoSA and CHARLS, while the dominant family size was two in HRS, ELSA, and SHARE. A relatively lower education level was found in the middle-aged and older population in CHARLS and MHAS, with the proportion of tertiary education being 1.31 and 9.58%, respectively. The proportion of currently employed individuals was among the highest in CHARLS (70.47%). A relatively higher prevalence of smoking (combined prevalence of former and current smoking) was seen in HRS (55.49%) and ELSA (62.16%), and a high frequency of alcohol drinking was seen in ELSA (86.61%) and SHARE (75.22%). Individuals in KLoSA and MHAS engaged in relatively fewer physical activities than their counterparts in other cohorts, with proportions of 66.10 and 64.95% never engaging in physical activities, respectively. Obesity was less prevalent in KLoSA (1.26%) and CHARLS (4.29%).

### Associations with individual risk factors

As shown in Table 2, multiple groups of risk factors were found to be associated with the prevalence of CVD among the middle-aged and older population in all cohorts. With respect to the demographic status, female sex was negatively associated with the CVD prevalence (except for CHARLS, with the odds ratio of 1.06), whereas age was positively associated in all settings (*p* < 0.001 for all).

**Table 2:** Factors associated with prevalence of cardiovascular disease by cohort (country/region).

	HRS*	**ELSA**	**SHARE**	**KLoSA**	**CHARLS**	**MHAS**
**Fixed Effects**
**Demographic characteristic**
Female gender (reference=male)	0.94 (0.93, 0.95) ^***^	0.94 (0.93, 0.96) ^***^	0.92 (0.92, 0.93) ^***^	0.95 (0.94, 0.97) ^***^	1.06 (1.04, 1.07) ^***^	0.98 (0.97, 0.99) ^***^
Age ≥70 years at interview (reference= < 70 years)	1.08 (1.07, 1.08) ^***^	1.04 (1.04, 1.05) ^***^	1.08 (1.07, 1.08) ^***^	1.02 (1.01, 1.03) ^***^	1.04 (1.03, 1.06) ^***^	1.03 (1.02, 1.04) ^***^
**Social connection**
Marital status (reference = married)
Living alone	1.02 (1.02, 1.03) ^***^	1.02 (1.01, 1.04) ^*^	1.04 (1.03, 1.04) ^***^	1.02 (1.01, 1.03) ^***^	1.03 (1.01, 1.04) ^**^	1.00 (0.99, 1.02)
Never married	0.98 (0.97, 1.00) ^*^	0.99 (0.96, 1.02)	0.98 (0.97, 1.00) ^**^	0.97 (0.91, 1.03)	1.00 (0.94, 1.06)	0.99 (0.96, 1.01)
Family Size (number of people living in household, reference = 1)	
2	1.00 (0.99, 1.00)	0.99 (0.97, 1.01)	1.00 (0.99, 1.00)	1.02 (0.99, 1.04)	1.00 (0.98, 1.01)	1.00 (0.99, 1.02)
≥3	0.99 (0.99, 1.00) ^*^	0.99 (0.98, 1.01)	0.98 (0.97, 0.99) ^***^	0.98 (0.96, 1.01)	0.99 (0.97, 1.01)	1.00 (0.99, 1.01)
**Socioeconomic Position**
Education level (reference = primary)
Upper secondary & vocational	1.00 (0.99, 1.02)	0.97 (0.95, 0.99) ^***^	0.97 (0.97, 0.98) ^***^	0.96 (0.95, 0.98) ^***^	1.03 (1.01, 1.05) ^*^	1.00 (0.97, 1.03)
Tertiary	0.97 (0.96, 0.99) ^***^	0.95 (0.93, 0.98) ^***^	0.97 (0.96, 0.97) ^***^	0.96 (0.94, 0.99) ^**^	1.05 (1.01, 1.10) ^*^	1.01 (0.99, 1.02)
Currently working for pay (reference= not working)	0.96 (0.95, 0.96) ^***^	0.97 (0.96, 0.98) ^***^	0.97 (0.96, 0.97) ^***^	0.98 (0.97, 0.98) ^***^	0.96 (0.95, 0.96) ^***^	0.97 (0.96, 0.99) ^***^
Household income level (reference =lower tertile)
Intermediate tertile	1.00 (0.99, 1.00)	1.01 (1.00, 1.02)	1.01 (1.00, 1.01) ^**^	0.99 (0.98, 0.99) ^***^	1.00 (0.99, 1.00)	1.01 (1.00, 1.02)
Highest tertile	0.99 (0.98, 0.99) ^***^	1.00 (0.99, 1.01)	1.01 (1.00, 1.01) ^**^	0.99 (0.99, 1.00) ^***^	0.98 (0.97, 0.99) ^***^	1.00 (0.99, 1.02)
**Behavioral factors**
Smoking (reference = non-smoking)
Currently smoking	1.02 (1.01, 1.03) ^***^	0.98 (0.96, 1.00) ^*^	1.00 (0.99, 1.01)	1.00 (0.98, 1.01)	1.02 (1.00, 1.03) ^**^	1.00 (0.98, 1.01)
Former	1.07 (1.06, 1.08) ^***^	1.02 (1.00, 1.03) ^*^	1.04 (1.03, 1.05) ^***^	1.03 (1.02, 1.05) ^***^	1.07 (1.05, 1.08) ^***^	1.02 (1.00, 1.03) ^*^
Alcohol Drinking (reference = not drinking)	0.97 (0.97, 0.97) ^***^	0.97 (0.96, 0.98) ^***^	0.99 (0.98, 0.99) ^***^	0.94 (0.94, 0.95) ^***^	0.99 (0.98, 1.00) ^***^	0.98 (0.97, 0.99) ^***^
physical activity level (reference = not engaged in physical activity)
1–3 per month	0.97 (0.96, 0.97) ^***^	0.95 (0.94, 0.96) ^***^	0.95 (0.95, 0.96) ^***^	0.99 (0.99, 1.00) ^**^	0.99 (0.97, 1.02)	0.98 (0.97, 0.99) ^***^
≥1 per week	0.96 (0.96, 0.97) ^***^	0.95 (0.94, 0.96) ^***^	0.93 (0.93, 0.94) ^***^	NA	0.98 (0.97, 0.99) ^***^	NA
**Physiological risk factors**
Obesity (reference = normal weight)
Overweight	0.99 (0.98, 0.99) ^***^	1.00 (0.99, 1.01)	0.99 (0.99, 1.00) ^**^	1.00 (1.00, 1.01)	1.01 (1.01, 1.02) ^*^	1.00 (0.99, 1.01)
Obesity	0.98 (0.98, 0.99) ^***^	1.00 (0.99, 1.01)	1.01 (1.00, 1.01) ^**^	0.99 (0.97, 1.01)	1.05 (1.03, 1.07) ^***^	1.01 (0.99, 1.02)
Presence of hypertension (reference = no history of hypertension)	1.10 (1.10, 1.11) ^***^	1.09 (1.08, 1.10) ^***^	1.08 (1.08, 1.08) ^***^	1.07 (1.06, 1.08) ^***^	1.13 (1.12, 1.14) ^***^	1.10 (1.09, 1.11) ^***^
Presence of diabetes (reference = no history of diabetes)	1.04 (1.04, 1.05) ^***^	1.06 (1.04, 1.08) ^***^	1.08 (1.07, 1.09) ^***^	1.03 (1.02, 1.04) ^***^	1.12 (1.10, 1.13) ^***^	1.02 (1.01, 1.03) ^***^
Wave (reference = Wave 1)
Wave 2	1.02 (1.02, 1.03) ^***^	1.02 (1.01, 1.03) ^***^	1.02 (1.02, 1.03) ^***^	1.01 (1.01, 1.02) ^***^	1.01 (1.00, 1.02) ^**^	1.00 (0.99, 1.01)
Wave 3	1.04 (1.04, 1.05) ^***^	1.05 (1.04, 1.05) ^***^	1.05 (1.05, 1.05) ^***^	1.02 (1.02, 1.03) ^***^	1.05 (1.04, 1.06) ^***^	NA
Wave 4	1.07 (1.06, 1.07) ^***^	1.08 (1.07, 1.09) ^***^	1.07 (1.07, 1.08) ^***^	1.03 (1.03, 1.04) ^***^	1.11 (1.10, 1.12) ^***^	NA
Wave 5	1.08 (1.08, 1.09) ^***^	1.11 (1.10, 1.11) ^***^	NA	1.04 (1.04, 1.05) ^***^	NA	NA
**Intercept**	1.26 (1.23, 1.29) ^***^	1.34 (1.29, 1.40) ^***^	1.25 (1.22, 1.27) ^***^	1.19 (1.14, 1.24) ^***^	1.00 (0.96, 1.04)	1.06 (1.03, 1.10) ^***^
**Random Effects**
Individual level	1.16 (1.16, 1.17)	1.15 (1.14, 1.15)	1.12 (1.12, 1.12)	1.09 (1.09, 1.10)	1.11 (1.11, 1.12)	1.03 (1.03, 1.04)
Residual	1.03 (1.03, 1.03)	1.03 (1.03, 1.03)	1.03 (1.03, 1.03)	1.02 (1.02, 1.02)	1.04 (1.04, 1.04)	1.06 (1.05, 1.06)

Data are presented as odds ratio (95% confidence interval). HRS, Health and Retirement Study; ELSA, English Longitudinal Study of Aging; SHARE, Survey of Health, Aging and Retirement in Europe; KLoSA, Korean Longitudinal Study of Aging; CHARLS, China Health and Retirement Longitudinal Study; MHAS, Mexican Health and Aging Study; NA, not available.

* *p* < 0.05.

** *p* < 0.01.

*** *p* < 0.001.

Social connection factors also mattered. Those living alone (compared with married/partnered) tended to have a higher CVD prevalence, with the ORs ranging from 1.00 in MHAS to 1.04 in SHARE. Those with a larger family size (≥3) had a significantly lower CVD prevalence in HRS (OR = 0.99) and SHARE (OR = 0.98).

In terms of socioeconomic position, we found that a higher education level, higher household income, and being employed were associated with a lower CVD prevalence in middle-aged and older individuals, although there were some exceptions as follows. Higher household income was significantly associated with a higher CVD prevalence in SHARE (OR = 1.01 in intermediate tertile and 1.01 in highest tertile), and a higher education level was associated with a higher CVD prevalence in CHARLS (OR = 1.03 in upper secondary and vocational education group and 1.05 in tertiary education group). There were also several non-significant associations with the education level in MHAS and the household income level in ELSA and MHAS.

Behavioral risk factors also played roles in the CVD prevalence. Current or former smoking, never drinking alcohol, and never engaging in moderate to vigorous physical activities were positively associated with the CVD prevalence. The significant ORs of current smoking, former smoking, alcohol drinking, engaging in physical activities one to three times per month, and engaging in physical activities more than once per week ranged from 0.98 in ELSA to 1.02 in HRS, from 1.02 in MHAS to 1.07 in HRS, from 0.94 in KLoSA to 0.99 in CHARLS, from 0.95 in ELSA to 0.99 in KLoSA, and from 0.93 in SHARE to 0.98 in CHARLS, respectively.

In terms of physiological risk factors, having been diagnosed with hypertension or diabetes was positively associated with the CVD prevalence in the full models, with ORs of 1.10 and 1.04 in HRS, 1.09 and 1.06 in ELSA, 1.08 and 1.08 in SHARE, 1.07 and 1.03 in KLoSA, 1.13 and 1.12 in CHARLS, and 1.10 and 1.02 in MHAS, respectively. A conflicting impact of obesity was observed among the different cohorts. Both overweight and obesity were associated with a higher CVD prevalence in CHARLS, with ORs of 1.01 and 1.05, respectively; however, they were associated with a lower CVD prevalence in HRS, with coefficients of 0.99 and 0.98, respectively.

### Relative contributions of risk factor groups

Table 3 shows the results of the dominance analyses. Physiological factors were the main driver of the CVD prevalence in the middle-aged and older population. In developing countries and regions, physiological factors accounted for a high share of the predicted variance (as high as 72.8% in MHAS and 57.5% in CHARLS). In Western developed countries and regions, the relative contributions were much lower (dominance of 27.5% in HRS, 27.7% in ELSA, and 29.8% in SHARE). In the KLoSA cohort, the dominance of physiological factors was 37.3%.

**Table 3:** Dominance of factors by cohort.

		**Gender**	**Age**	**Social connection**	**Socioeconomic position**	**Behavioral factors**	**Physiological risk factors**
HRS	2010	4.7	25.2	3.2	19.7	19.4	27.9
	2012	5.3	23.1	3.6	19.9	22.8	25.3
	2014	4.9	23.2	2.9	21.8	21.7	25.6
	2016	2.9	24.6	4.0	24.4	18.3	25.8
	2018	4.2	23.4	3.9	23.4	17.7	27.5
ELSA	2010	2.6	28.4	4.4	20.6	24.2	19.8
	2012	3.3	26.0	4.4	18.0	28.5	19.8
	2014	3.4	26.3	3.2	19.2	26.0	21.9
	2016	6.5	24.4	3.1	15.3	27.7	23.0
	2018	5.3	21.8	3.5	15.5	26.1	27.7
SHARE	2011	6.9	28.0	3.5	18.6	19.6	23.5
	2013	8.0	26.8	4.0	16.7	18.7	25.8
	2015	6.9	25.7	3.7	15.3	19.3	29.1
	2017	8.3	25.5	3.2	15.5	17.7	29.8
KLoSA	2011	2.0	9.4	3.6	23.3	18.7	43.0
	2013	2.1	10.4	4.8	26.6	15.8	40.3
	2015	1.4	14.7	6.1	23.0	14.9	39.9
	2017	1.7	16.6	6.0	22.3	16.3	37.1
	2019	1.9	18.8	7.0	20.0	14.9	37.3
CHARLS	2011	1.1	2.7	2.5	21.8	9.0	62.9
	2013	0.8	1.7	1.0	21.7	9.3	65.5
	2015	3.3	3.7	3.4	15.5	9.5	64.5
	2018	2.6	4.9	3.0	18.4	13.6	57.5
MHAS	2015	1.4	7.2	2.0	8.2	13.8	67.5
	2018	1.0	10.4	0.7	9.4	5.7	72.8

Data are presented as standardized dominance statistic (%). HRS, Health and Retirement Study; ELSA, English Longitudinal Study of Aging; SHARE, Survey of Health, Aging and Retirement in Europe; KLoSA, Korean Longitudinal Study of Aging; CHARLS, China Health and Retirement Longitudinal Study; MHAS, Mexican Health and Aging Study.

The set of behavioral factors was one of the top drivers of the CVD prevalence, with opposite rankings across cohorts. In Western developed countries and regions, behavioral factors highly contributed to the CVD prevalence (as high as 17.7% in HRS, 26.1% in ELSA, and 17.7% in SHARE). The proportions were lower in cohorts in developing regions, including CHARLS (13.6%) and MHAS (5.7%). The dominance of behavioral factors in KLoSA was 14.9%.

Socioeconomic position was highly associated with the CVD prevalence in the middle-aged and older population of all cohorts, with dominance of 23.4% in HRS, 15.5% in ELSA, 15.5% in SHARE, 20.0% in KLoSA, 18.4% in CHARLS, and 9.4% in MHAS. Age also accounted for a high share of predicted variance, with dominance proportions of 23.4% in HRS, 21.8% in ELSA, 25.5% in SHARE, 18.8% in KLoSA, 4.9% in CHARLS, and 10.4% in MHAS.

**Figure 1:**
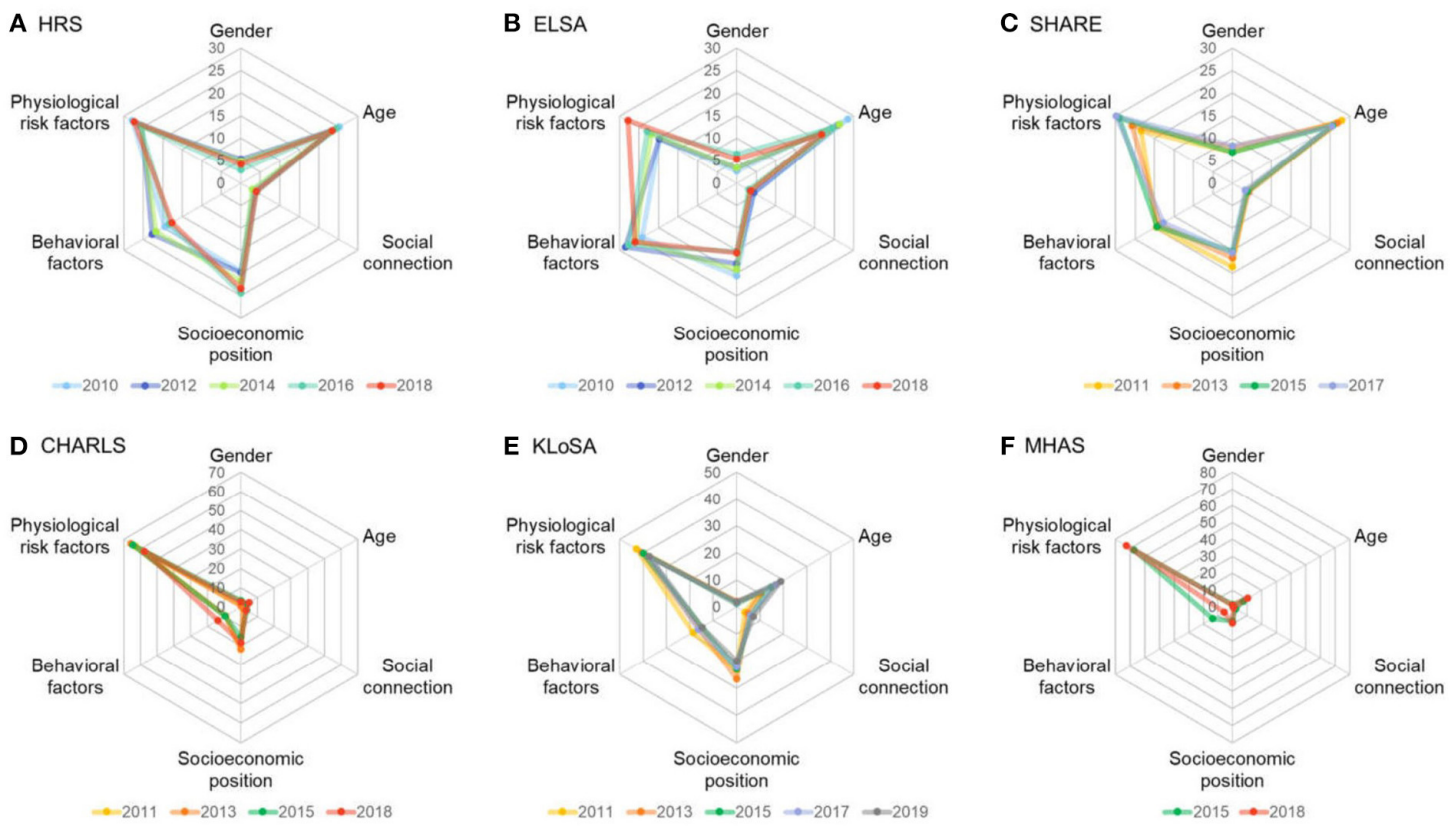
Contributions of the cardiovascular disease determinants by cohort. Data are presented as standardized dominance statistic (%). HRS, Health and Retirement Study; ELSA, English Longitudinal Study of Aging; SHARE, Survey of Health, Aging and Retirement in Europe; KLoSA, Korean Longitudinal Study of Aging; CHARLS, China Health and Retirement Longitudinal Study; MHAS, Mexican Health and Aging Study.

The comparison of risk factor contribution patterns is shown in Figure 1. Although physiological factors were the major contributor to the CVD prevalence in the middle-aged and older population in all cohorts, the contributions were much higher in developing countries (CHARLS and MHAS). In Western developed countries and regions (HRS, ELSA, and SHARE), behavioral factors had very high contributions to the CVD prevalence. In the KLoSA cohort, where the level of economic development fell in the middle among all cohorts, the dominance proportion of the physiological factors and behavioral factors also showed a transitional trend between the two levels mentioned above.

## Discussion

To the best of our knowledge, this is the first study to investigate the contributions of social, behavioral, and physiological determinants to the CVD prevalence in the middle-aged and older population. Although several determinants of CVD were well documented in prior studies, little research has been conducted on the contribution of each determinant in the same setting. With the growing burden of CVD and its widening disparities across countries and regions during the past decade, social determinants of health have been systematically considered to capitalize on CVD prevention and control strategies. However, different settings are faced with challenges introduced by different risk factors, the contributions of which can vary across developed and developing countries. By taking advantage of multi-country population-based cohorts, we were able to demonstrate the different patterns of determinant contributions across different countries and regions, highlighting the importance of national context in the CVD prevalence.

In the middle-aged and older population of our harmonized cohorts, we found that the prevalence of CVD was similar to that in previous studies of sample countries (16–18) but slightly lower than the national estimated prevalence (19). Nevertheless, we focused on confirming that five specific groups of risk factors (demographic characteristics, social connection, socioeconomic position, behavioral factors, and physiological risk factors) had significant impacts on the CVD prevalence in the middle-aged and older population, as indicated by prior studies (20). The association between demographic factors and the CVD prevalence is well documented. Age dominates the CVD risk factors (21–23), mainly because of the summed effects of prolonged exposure to other modifiable risk factors (24). Social connection is also important (especially the marital status as shown by a recent systematic review and meta-analysis (25)) through the mechanisms of spousal support (26), stress (27, 28), and selection theories (29). The impact of socioeconomic position on the risk of CVD has also been validated, although the context is important (8, 10, 30). Education has long been cited as a risk factor for CVD (7, 31). Behavioral factors are undoubtedly essential to the CVD prevalence, with the main drivers being an unhealthy diet, physical inactivity, tobacco use, and harmful use of alcohol (32–34). In terms of physiological risk factors, hypertension and diabetes are among the most important risk factors for CVD (33, 35), whereas obesity contributes both directly and indirectly to the CVD risk (36). Understanding the links of multiple groups of risk factors with the CVD prevalence can help to design public health interventions and policies for the neediest countries and regions.

Among all the determinants in the present study, physiological factors contributed the most in all cohorts, but the relative contribution was higher in the developing countries. In the developed countries, although the prevalence of obesity, hypertension, and diabetes was higher among the older population, the contribution of these factors to individual CVD variances was relatively low. This phenomenon may be largely due to the fact that chronic conditions were mostly under control by the well-functioning healthcare systems in developed countries (37), in which healthcare services such as pharmacological treatments, behavioral intervention, and environment support for chronic disease management are of high quality and homogeneity. In developed settings, the CVD mortality rate is relatively lower although the prevalence is higher than that in less-developed countries, and the national capacity of non-communicable disease control is reportedly an important reason for this (38). Most healthcare services for CVD prevention and control are included in universal health coverage packages in these countries, especially for the middle-aged and older population. Thus, these chronic conditions can be controlled more effectively with lifestyle interventions and more frequent use of proven pharmacologic therapies and revascularization (39), through which the incidence of secondary diseases such as CVD can be reduced, and the difference among populations is relatively small. Age, behavioral factors, and other risk factors may therefore have a relatively higher contributions in developed countries and regions, as shown by our results. In South Korea, which is undergoing both societal and epidemiological changes, we observed a relatively larger contribution of socioeconomic position and a smaller contribution of behavioral factors. Additionally, in line with recent studies, we recognized the potential coexistence of multiple chronic diseases/conditions among the older population, which may shift our current knowledge on single chronic diseases. Therefore, further studies must focus on the prevalence and cluster patterns of multiple chronic diseases to help better explain the roles of physiological factors.

We explored the potential mechanisms underlying the relatively high contribution of the socioeconomic position and behavioral factors to the CVD prevalence in the middle-aged and older population. The important roles of the health system may explain why the contribution of socioeconomic factors was higher in developing than developed countries because these factors hugely influence health literacy and access to healthcare services (40, 41). Intensive interactions between patients with chronic conditions and their care managers were reported in developed regions (42); this is a good example of improved management of chronic conditions under generally higher-quality healthcare systems and reduced disparity caused by individual socioeconomic factors. Global disparities in access to CVD care have resulted in an obvious hypertension and diabetes care cascade in developing countries. A study in 2017 showed that nearly half of Chinese adults aged 35 to 75 years had hypertension, but fewer than one-third were being treated and fewer than 1 in 12 had well-controlled blood pressure (43). Compared with China, the proportion of control in the United States (43.5%) was much higher (44). The situation was similar for patients with diabetes (45). The importance of behavioral factors is evident in Mexico, where access and quality of care are relatively low (46). Strengthening primary healthcare has been proven to be a cost-effective approach to address the cascade, but progress has stalled because of the complexity of horizontal interventions.

Considering ethnic, cultural, and context-related matters in determining the risk factors for disease as well as the comparative effectiveness of interventions (47), actions aiming to prevent and control the global CVD burden may need to tailor their approaches to different countries. In developed countries, reducing individual risk behaviors may be more effective in lowering the CVD burden, while in developing countries, strengthening the healthcare system may have higher priority. Moreover, joint international projects may help bridge the communication between developing and developed countries, which will facilitate sharing not only theoretical but also practical experiences of enhancing the capacity of healthcare systems.

This study had several limitations. First, the observational nature of our study limited our ability to investigate the causal relationship between risk factors and the CVD prevalence. Rather, the longitudinal associations found in the present study underscore the need for research to capitalize on the mechanistic basis behind the observed link between several social and environmental risk factors and inequalities in the CVD prevalence. Second, because of limitations in data availability, some other contributory factors (such as dietary factors) may have been excluded from this analysis, and these factors may vary across counties. Further studies will need to focus on dietary factors and their effects on the CVD prevalence among countries. Third, we attempted to identify differences in risk factors for CVD among different countries and regions with different social contexts. It was difficult to establish the parameters of the research. However, the methods for statistical analysis of data inherent in this study still contributed to the establishment of correlations between risk factors and the CVD prevalence in comparative studies and helped to provide implications for reducing the inequalities in the CVD prevalence by tacking with different risk factors in different settings.

## Conclusion

The present study demonstrated the different patterns of determinant contributions across settings, highlighting the importance of national context in the CVD prevalence. Faced with the challenges produced by different risk factors, the implementation of tailored CVD prevention and control strategies may help to narrow disparities in the CVD prevalence by promoting health management and enhancing the capacity of healthcare systems across different countries.

## Data availability statement

Publicly available datasets were analyzed in this study. This data can be found here: https://g2aging.org/.

## Ethics statement

Ethical review and approval was not required for the study on human participants in accordance with the local legislation and institutional requirements. Written informed consent for participation was not required for this study in accordance with the national legislation and the institutional requirements.

## Author contributions

JZ conceived the study, analyzed the data, and interpreted the results. YF interpreted the results and drafted the manuscript. YY, YZ, DY, and MS contributed to the study design and results interpretation. YJ and Z-JZ conceived and supervised the study. All authors critically reviewed the manuscript and approved it.

## Funding

This paper was supported by the National Natural Science Foundation of China (No. 71904004), the Clinical Medicine Plus X - Young Scholars Project, Peking University, and the Fundamental Research Funds for the Central Universities (No. PKU2022LCXQ008).

## Conflict of interest

The authors declare that the research was conducted in the absence of any commercial or financial relationships that could be construed as a potential conflict of interest.

## Publisher's note

All claims expressed in this article are solely those of the authors and do not necessarily represent those of their affiliated organizations, or those of the publisher, the editors and the reviewers. Any product that may be evaluated in this article, or claim that may be made by its manufacturer, is not guaranteed or endorsed by the publisher.
