# Supplementary material for: Disparities in cardiovascular disease prevalence among middle-aged and older adults: Roles of socioeconomic position, social connection, and behavioral and physiological risk factors
Source: Front Cardiovasc Med. 2022 Oct 14;9:972683. doi: 10.3389/fcvm.2022.972683 (PMC9614039; doi:10.3389/fcvm.2022.972683)
Supplement: Supplementary file 1 [file Table_1.DOCX]

**Supplementary Information**

**Disparities in Cardiovascular Diseases Prevalence Among Middle-Aged and Older Adults: Role of Socioeconomic Position, Social Connection, Behavioral and Physiological Risk Factors**

**Ji Zhang, Yian Fang, Yao Yao, Yang Zhao, Dahai Yue, Meekang Sung, Yinzi Jin, Zhi-Jie Zheng**

Contents

Supplement Table 1. Final sample size of each cohort-------------------------------------------------Page 2

Supplement Table 2. Definition and concordance of outcome variable across cohorts------------Page 3

Supplement Table 3. Definition and concordance of explanatory variables across cohorts-------Page 4

| **Supplement Table 1**. Final sample size of each cohort | | | | |
| --- | --- | --- | --- | --- |
|  | Wave | Men | Women | Total |
| HRS | 2010 | 8767 | 11799 | 20566 |
|  | 2012 | 8205 | 11298 | 19503 |
|  | 2014 | 7410 | 10485 | 17895 |
|  | 2016 | 8280 | 11371 | 19651 |
|  | 2018 | 6774 | 9556 | 16330 |
| ELSA | 2010 | 3404 | 4023 | 7427 |
|  | 2012 | 3591 | 4264 | 7855 |
|  | 2014 | 3058 | 3690 | 6748 |
|  | 2016 | 2852 | 3481 | 6333 |
|  | 2018 | 2525 | 3075 | 5600 |
| SHARE | 2011 | 13387 | 15235 | 28622 |
|  | 2013 | 16629 | 17486 | 34115 |
|  | 2015 | 15940 | 19841 | 35781 |
|  | 2017 | 13640 | 17542 | 31182 |
| KLoSA | 2011 | 3304 | 4344 | 7648 |
|  | 2013 | 3214 | 4270 | 7484 |
|  | 2015 | 3420 | 4528 | 7948 |
|  | 2017 | 3188 | 4297 | 7485 |
|  | 2019 | 2938 | 4000 | 6938 |
| CHARLS | 2011 | 3965 | 4223 | 8188 |
|  | 2013 | 1971 | 3469 | 5440 |
|  | 2015 | 4896 | 4926 | 9822 |
|  | 2018 | 6185 | 6156 | 12341 |
| MHAS | 2015 | 5147 | 7074 | 12221 |
|  | 2018 | 2306 | 3957 | 6263 |

| **Supplement Table 2**. Definition and concordance of outcome variable across cohorts | | |
| --- | --- | --- |
|  | Ever had heart problem? | Ever had stroke? |
|  | (Has a doctor ever told you that you have had a…) | (Has a doctor ever told you that you have had a…) |
| HRS | heart attack, coronary heart disease, angina, congestive heart failure, or other heart problems | stroke |
| SHARE | heart attack, including myocardial infarction or coronary thrombosis, or any other heart problem, including congestive heart failure | cerebral vascular disease |
| ELSA | angina, a heart attack (including myocardial infarction or coronary thrombosis), congestive heart failure, a heart murmur, an abnormal heart rhythm, or any other heart trouble | cerebrovascular disease |
| KLoSA | heart attack, coronary heart disease, angina, congestive heart failure | possible ischemic attack |
| CHARLS | heart attack, coronary heart disease, angina, congestive heart failure, or other heart problems | stroke |
| MHAS | heart failure, cardiac failure, congestive heart failure, arrhythmia, angina, or a heart attack | stroke |

| **Supplement Table 3.** Definition and concordance of the explanatory variables across cohorts | | |
| --- | --- | --- |
| Variables | Definition and Concordance | Scales |
| **Social Connection** | | |
| Marital status | Married and partnered were combined or separated in harmonized data, we combined them in this study, so as divorced/widowed/separated. | 1. Married/partnered 2. Living alone 3. Never married |
| Family Size | Number of residents living in the household.  *Baseline data (in wave 1) were used in KLoSA. | 1. 1 2. 2 3. >2 |
| **Socioeconomic position** | | |
| Education level | Harmonized education scale is a simplified version of 1997 International Standard Classification of Education (ISCED-97) codes. | 1. Less than lower secondary 2. Upper secondary & vocational 3. Tertiary |
| Employment | The time period varied across studies. | 1. Not working 2. Currently working for pay |
| Household income level | Total household income, before or after tax in harmonized data. We divided all household into three groups based on the total income in each study. | 1. Lower tertile 2. Intermediate tertile 3. Highest tertile |
| **Behavioral Factors** | | |
| Smoking | This variable is derived by combining two variables: whether the respondents ever smoked cigarettes, and whether the respondents smokes now. | 1. Never 2. Currently 3. Formerly smoking |
| Alcohol Drinking | Whether the respondent ever drinks alcohol beverages· The time period varied across studies. | 1. Never 2. Ever drinking alcohol in a specific period |
| Frequency of moderate to vigorous physical activity | Frequencies of moderate and Vigorous physical activity were combined in HRS, SHARE, ELSA, and CHARLS· Only vigorous physical activity were included in analyses in KLoSA and MHAS. | 1. Never 2. 1-3 per month 3. >=1 per week (including 'everyday') |
|  | The scales varied across studies |  |
| **Physiological risk factors** | | |
| Obesity/BMI | Measured or self-reported body mass weight and height were obtained, and the body mass index were calculated as weight divided by the square of height. Self-reported: HRS, KLoSA, and SHARE; Measured: ELSA (r4, r6, r8), CHARLS, and MHAS. | 0. Normal weight: BMI<25 1. Overweight: 25<=BMI<30;  2. Obesity: BMI>=30· |
| History of hypertension | Self-reported history, imputations were made according to former waves (if diagnosed in wave N, then diagnosed in following waves). | 1. No 2. Yes |
| History of diabetes | Self-reported history, imputations were made as above· | 1. No 2. Yes |
